# On Elective Elimination by the Salivary and Other Secretions

**Published:** 1854-01

**Authors:** M. Cl. Bernard


### ARTICLE XIX.

### *On Elective Elimination by the Salivary and other Secretions.* By M. CL. BERNARD.

IN this paper Mr. Bernard calls attention to the fact, that some of the secretions rapidly eliminate certain substances, while other substances, equally soluble, are either eliminated much more slowly, or not at all. He relates the result of a series of experiments, in which iodide of potassium, iodide of iron, lactate of iron, cane and grape sugar, and yellow prussiate of potass, were injected into the veins, and the various secretions then tested for their presence.

Of these, *iodide of potassium* appeared, at latest, in from 30 to 40 seconds in the saliva, and was also rapidly observed in the tears and pancreatic juice. It required more than an hour to become detectible in the urine or the bile; and if injected in very small quantities, was not found in these at all. Introduced into the stomach, and especially fasting, it was found in the saliva in 1½ minute. The *yellow prussiate of potass* was not discernible in the saliva, while in seven minutes it was found in the urine and abundantly eliminated; the serum of the blood also exhibiting a notable quantity in an hour and a half. It was also found in the bile, while, although it was thus circulating in the blood, no traces of it could be found in the pancreatic juice. *Grape and cane sugar* never passed into the saliva or pancreatic fluid, while it was manifested in the bile and urine, though less rapidly than the prussiate. As various authors state they have detected sugar in the saliva in *diabetes*, the author examined that of several such patients under M. Rayer's care. In none was sugar detected, although the bronchial mucus and sputa evidently contained it. The *mammary gland*, which in the normal state contains the sugar of milk in its secretion, refused passage to grape or cane sugar, even when these substances existed in large quantities in the blood. A saturated solution of *lactate of iron*, thrown into the veins, never gives rise to iron in the saliva; but when the iron is injected as an *iodide*, it obtains admission into the saliva, both the iron and the iodine being then detectible.

This expulsion of certain salts by this or that secretion, is not the only peculiarity the history of elimination presents. Some substances are eliminated rapidly and completely, while others remain within the tissues for a more or less long period. It is well known that certain of these, as mercury, antimony and arsenic, become localized in certain organs—*e. g.*, the liver—and are then gradually eliminated; but it has not been noted that others, as the iodide of potassium, which are perfectly soluble, and remains soluble in the economy, wherein they circulate without enduring any accident, may remain for a certain time in the substance of the organs. Two or three weeks after iodide of potassium had been introduced into the stomach of several dogs, and long after its supposed entire elimination by the urine, in which it had ceased to appear, it was found in the saliva and gastric juice. If, however, purgatives were employed after administering the iodine, it ceased to be detectible in a few days in any of the secretions.—*Archives Générales*, N. S. vol. i, p. 5.—*Brit. and For. Medico-Chirurgical Review.*

